# Effect of Robotic-Assisted Gait at Different Levels of Guidance and Body Weight Support on Lower Limb Joint Kinematics and Coordination

**DOI:** 10.3390/s23218800

**Published:** 2023-10-29

**Authors:** Yosra Cherni, Yoann Blache, Mickael Begon, Laurent Ballaz, Fabien Dal Maso

**Affiliations:** 1 Laboratoire de Simulation et Modélisation du Mouvement, École de Kinésiologie et des Sciences de l’Activité Physique, Université de Montréal, Montréal, QC H3T 1J4, Canada; 2 Centre de Recherche du CHU Ste Justine, Montréal, QC H3T 1C5, Canada; 3 Centre de Recherche et d’Innovation Sur le Sport, Université de Lyon, 69007 Lyon, France; 4 Département des Sciences de l’Activité Physique, Université du Québec à Montréal, Montréal, QC H2L 2C4, Canada; 5 Centre Interdisciplinaire sur le Cerveau et l’Apprentissage, Université de Montréal, Montréal, QC H3C 3J7, Canada

**Keywords:** Lokomat, treadmill, gait pattern, angular velocity, continuous relative phase, statistical parametric mapping, body weight support, robotic assistance

## Abstract

The Lokomat provides task-oriented therapy for patients with gait disorders. This robotic technology drives the lower limbs in the sagittal plane. However, normative gait also involves motions in the coronal and transverse planes. This study aimed to compare the Lokomat with Treadmill gait through three-dimensional (3D)-joint kinematics and inter-joint coordination. Lower limb kinematics was recorded in 18 healthy participants who walked at 3 km/h on a Treadmill or in a Lokomat with nine combinations of Guidance (30%, 50%, 70%) and bodyweight support (30%, 50%, 70%). Compared to the Treadmill, the Lokomat altered pelvic rotation, decreased pelvis obliquity and hip adduction, and increased ankle rotation. Moreover, the Lokomat resulted in significantly slower velocity at the hip, knee, and ankle flexion compared to the treadmill condition. Moderate to strong correlations were observed between the Treadmill and Lokomat conditions in terms of inter-joint coordination between hip–knee (r = 0.67–0.91), hip–ankle (r = 0.66–0.85), and knee–ankle (r = 0.90–0.95). This study showed that some gait determinants, such as pelvis obliquity, rotation, and hip adduction, are altered when walking with Lokomat in comparison to a Treadmill. Kinematic deviations induced by the Lokomat were most prominent at high levels of bodyweight support. Interestingly, different levels of Guidance did not affect gait kinematics. The present results can help therapists to adequately select settings during Lokomat therapy.

## 1. Introduction

Robotic-assisted gait orthoses provide task-oriented and mass-practice therapy in individuals with neuromotor disorders [1,2]. These devices aim to drive patients’ lower limbs in a physiological gait pattern. Patients can, therefore, benefit from long training sessions; more than 1000 steps can typically be achieved [3,4,5,6,7] as compared with a conventional overground training session during which usually fewer than 200 steps are achieved. Previous studies showed a greater improvement in overall ambulatory capacity [8,9,10] and dynamic balance [11,12] when robotic-assisted gait therapy is combined with conventional therapies than conventional therapies alone. The Lokomat is amongst the most used robotic-assisted gait orthoses in rehabilitation centers [1]. This technology assists patients to walk on a Treadmill using a harness that dynamically supports their body weight and bilateral motorized leg orthoses that drive their hips and knees in a predefined sagittal gait pattern. Both bodyweight support (BWS) and leg guidance are adjustable between 0% and 100%. There is some, albeit limited, evidence from clinical trials that Guidance and BWS Lokomat settings might be essential to increase the impact of this technology on the gait biomechanics in individuals with neuromotor disorders [13,14,15]. However, the existing literature has not yet sufficiently investigated how Lokomat settings influence lower limb gait patterns [16,17].

Although the Lokomat only drives patients’ lower limbs in the sagittal plane, individuals walking in the Lokomat showed trunk and pelvis translations and rotations in the coronal and transverse planes [2,18]. However, the range of motion of these movements differed compared to Treadmill gait and are affected by Lokomat settings. At different levels of Guidance (30%, 60%, and 100%) and BWS (0%, 30%, and 50%), Swinnen et al. [2,18] reported smaller lateral translations of the trunk and the pelvis, smaller anteroposterior flexion and axial rotation of the trunk and larger lateral flexion of the trunk during the Lokomat compared to Treadmill gait. Furthermore, the authors highlighted that an increase in BWS significantly reduced the range of motion of the thorax flexion and rotation in healthy adults [2]. In contrast, Guidance increase did not affect gait [2,18]. Concerning lower-limb kinematics, Hidler et al. [19] showed a greater hip and ankle range of motion in the sagittal plane in six healthy participants during Lokomat compared to Treadmill gait. However, to the best of our knowledge, no study investigated leg kinematics in the coronal and transverse planes during Lokomat walking. Furthermore, normative gait also involves a precise rhythmic pattern so that joint angular velocities [20] and inter-joint coordination, assessed by the continuous relative phase (CRP) [21,22], are fundamental gait parameters. However, the latter has not yet been studied during the Lokomat gait.

This exploratory study aimed to compare joint angles, angular velocities, and joint coordination during non-assisted Treadmill gait and assisted Lokomat gait with various levels of BWS and Guidance. As the Lokomat has been designed to drive the lower limbs in the sagittal plane, it was hypothesized that joint kinematics and coordination in this plane would present strong similarities between the Treadmill and Lokomat conditions. On the other hand, it is expected that joint kinematics would differ significantly between Treadmill and Lokomat conditions in the coronal and transverse planes. Finally, since only BWS affected the kinematics of the thorax and pelvis [2,18], we expected higher similarity scores between the Treadmill and Lokomat conditions with low percentages of BWS than with high percentage of BWS.

## 2. Materials and Methods

### 2.1. Participants

Eighteen right-footed healthy adults (9 females, age: 26.5 ± 4.7 years; height: 1.72 ± 0.09 m; mass: 66.1 ± 11.4 kg) volunteered for this study. Participants were excluded if they reported any lower limb and back injuries and/or orthopedic surgery at the time of the experiment or during the last year. All participants provided their written informed consent approved by the Ethics Committee of Sainte-Justine Hospital (#4049).

### 2.2. Protocol

**Figure 1:**
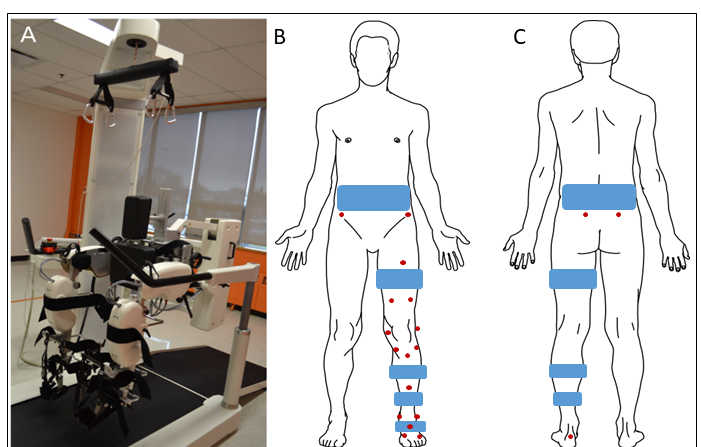
(**A**) Lokomat Pro version 6.0. (**B**) Front and (**C**) back schematic representations of a participant equipped with reflective markers (in red). Notes. During the static pose and functional movements, two markers were placed on the posterior superior iliac spines. These markers were removed during the experimental conditions as the Lokomat harness (blue) hid these bony landmarks.

The Lokomat Pro version 6.0 was used for this study (Figure 1A). The Lokomat is a robotic gait training system comprising a treadmill, a bodyweight support system, and four robotic actuators attached and adjusted to the participant’s legs. The robotic actuators integrated into the Lokomat guide hip and knee movements along the sagittal plane, while the ankle joint kinematics is supported by a spring system. During the experimentation, an experienced experimenter adjusted the hip and knee range of motion of the Lokomat actuators as well as the synchronization between the speed of the treadmill and the feet.

Familiarization session. Three to six days before the main session, the exoskeleton was adapted to the participants’ anthropometry (i.e., legs, cuffs and harness sizes, joint alignments). Participants were familiarized with the Lokomat gait during a 45-min active walk. They walked at various speeds (1.5 to 3.0 km/h), BWS (20% to 80%), and Guidance (20% to 80%) settings.

Main session. For the Lokomat conditions, each participant was asked to walk as normally as possible, following the movements generated by the Lokomat in nine different conditions that combined different settings of BWS (30%, 50%, 70%) and GF (30%, 50%, 70%). These levels of BWS and Guidance match the range of Lokomat settings reported during therapy [23,24,25,26,27]. The walking speed was set to 3 km/h for each participant. This speed was selected as the Lokomat best operates close to this walking speed [19,28]. Participants were provided with a 1–2 min familiarization period before the recording of each condition to ensure a stable gait pattern. Moreover, the order of the nine Lokomat conditions was randomized between participants. The duration of measurements was 60 s for each condition. A reference condition, consisting of treadmill walking with no assistance from the Lokomat, was recorded after the completion of the Lokomat conditions (n = 9). In order to limit possible after-effects due to the Lokomat walking conditions, each participant was asked to walk for 5 min on the treadmill, and only the 5th minute was recorded. Participants had a 5-min break before starting the treadmill condition. Participants interspersed short breaks between their sessions on the Lokomat and the Treadmill.

### 2.3. Data Recording

Marker trajectories were recorded using a 12-camera motion capture system (T-40 and T-20 Vicon cameras, Oxford Metrics Ltd., Oxford, UK) at a rate of 300 Hz. For Lokomat and Treadmill conditions, 16 skin markers were placed on the left and right foot (n = 4), shank (n = 6), thigh (n = 5), and the anterior superior iliac spine (n = 1) (Figure 1B,C) [29]. During the static pose and functional movements (described thereafter), two markers were added to the posterior superior iliac spines (Figure 1C). These markers were not present during the experimental conditions as the Lokomat harness hid these bony landmarks. The static pose, including the full marker set, was recorded after the Treadmill session. Participants held a standing posture (static pose) and performed hip and ankle flexion–extension, abduction–adduction, circumduction, and knee flexion–extension movements. These recordings were required to functionally locate hip and ankle joint centers of rotation and knee axes of rotation as described thereafter [30]. 

### 2.4. Data Processing

Joint kinematics reconstruction. Hip and ankle centers of rotation and knee axes of rotation were determined using functional methods [31,32]. Then, joint kinematics were obtained using an inverse kinematics procedure based on a nonlinear least squares algorithm [33]. The multibody kinematic model was composed of seven segments, namely, pelvis and left and right thigh, shank, and foot articulated by 20 degrees of freedom, namely, three rotations and three translations at the pelvis, three rotations at each hip, flexion–extension at each knee and three rotations at each ankle. Kinematic data were filtered with a zero-lag 10 Hz lowpass Butterworth filter. Only the kinematic of the right leg was reported for further analysis. Moreover, we did not consider hip rotation and ankle abduction-adduction as these degrees of freedom presented large variability.

Angular velocities. The angular velocities were calculated as the first derivative of joint angles. Additionally, as the walking speed was constant for all participants and for all conditions of the Lokomat and Treadmill, the stride rate was calculated to better interpret the results of the angular velocities.

Inter-joint coordination. The inter-joint coordination was evaluated with the continuous relative phase (CRP) method, which is the calculation of the phase angle difference between the two joints. The phase angle of each degree of freedom was computed using the Hilbert transform [34] before calculating the hip-knee, hip-ankle, and knee-ankle CRP.

Heel strikes. Gait events were determined and measured based on the trajectory of a marker positioned on the right heel. Heel strikes events were automatically determined using a custom-made script. This script determined a threshold that corresponded to the average ± 2 std of the heel marker height during the stance phase. A heel strike event was tagged when the heel marker height was below this threshold after the swing phase. The time histories of the joint angles, joint angular velocities, and CRP were time-normalized between two heel strike events.

### 2.5. Statistics

Four gait cycles in each experimental condition and each participant were considered for statistical analysis. Firstly, paired *t*-tests from the statistical parametric mapping package [35] were used to compare time histories of joint angles and velocities and CRP between each Lokomat (n = 9) and the Treadmill condition. Considering the primary purpose of this exploratory study to compare Treadmill and Lokomat gaits with no hypothesis formulated regarding specific variables, the significance threshold was set to 0.05 and kept uncorrected to appropriately maximize sensitivity. Only differences lasting more than 5% of the gait cycle were considered for analysis. Secondly, Pearson’s correlation analyses were used to assess the similarity of joint angles, angular velocities, and CRP between each Lokomat (n = 9) and the Treadmill condition. Correlations in the range of 0.0–0.3, 0.3–0.7, and 0.7–1.0 and their negative counterparts were considered weak, moderate, and strong correlations, respectively [36].

## 3. Results

### 3.1. Joint Angles

**Figure 2:**
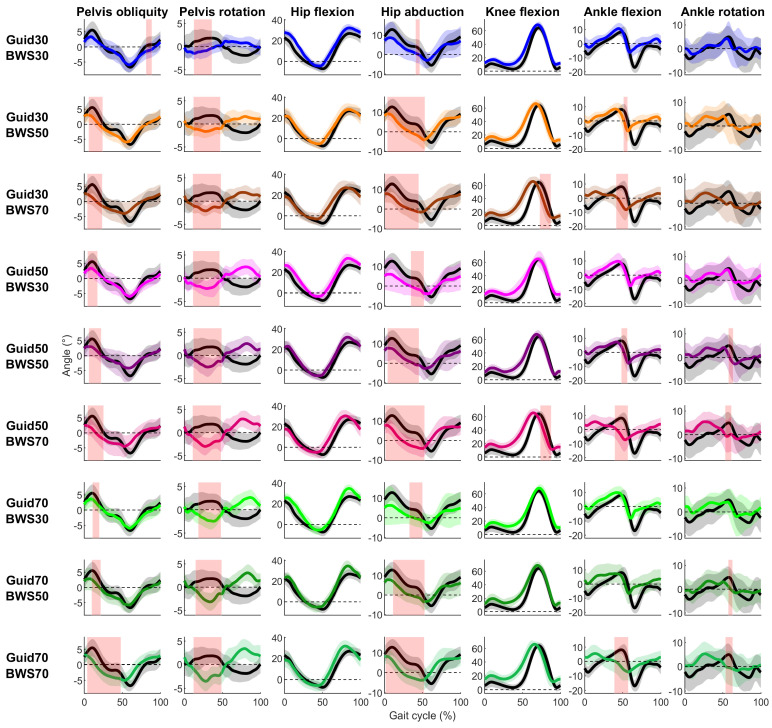
Participants’ mean (solid lines) ± standard deviation (shaded zones) joint angles during the Treadmill (black) and Lokomat (colored) conditions throughout the gait cycle for each degree of freedom (column) and Lokomat setting (row). Light red shaded zones report a significant difference between the Treadmill and Lokomat conditions (FDR *p*-values are reported in Table S1).

**Figure 3:**
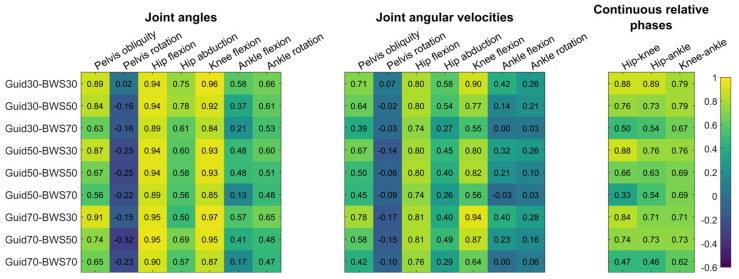
Heat map representation of correlation coefficients between the Treadmill and each Lokomat condition for joint angles, angular velocities, and continuous relative phases. Numbers represent correlation coefficients.

In the sagittal plane, the Lokomat condition presented few significant differences with the Treadmill condition (Figure 2, Table S1). For the hip flexion, there was no difference reported, and correlations were strong (0.88 ≤ R ≤ 0.97) (Figure 3). Knee and ankle showed significantly smaller flexion in the Lokomat than the Treadmill condition with moderate to strong (0.65 ≤ R ≤ 0.98) and low to moderate correlations (0.10 ≤ R ≤ 0.71), respectively. Significant differences lasted less than 20% of the gait cycle and occurred during the swing phase and the end of the stance phase for the knee and ankle, respectively.

In the coronal and transverse planes, the Lokomat conditions systematically reduced pelvis obliquity, hip adduction, pelvis, and ankle internal rotation compared to the Treadmill condition (Figure 2). Significant differences were reported during the stance phase for the pelvis obliquity, pelvis rotation, and hip adduction and around the toe-off period for the ankle internal rotation. The correlations were moderate to strong for pelvic obliquity (0.56 ≤ R ≤ 0.93), low for the pelvis rotation (−0.32 ≤ R ≤ 0.02), moderate for the hip abduction (0.43 ≤ R ≤ 0.73), and low to moderate for the ankle rotation (0.18 ≤ R ≤ 0.56) (Figure 3).

### 3.2. Joint Angular Velocities

**Figure 4:**
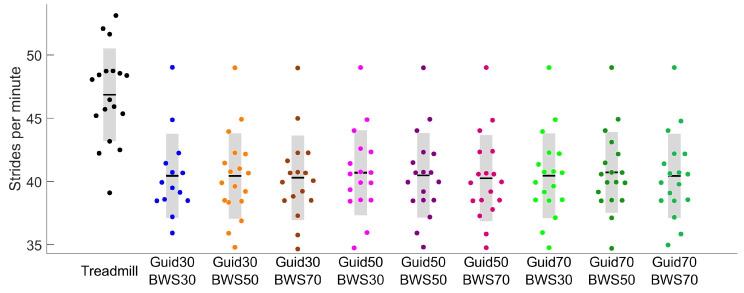
Stride rates in the Treadmill (black) and different Lokomat (colored) conditions.

**Figure 5:**
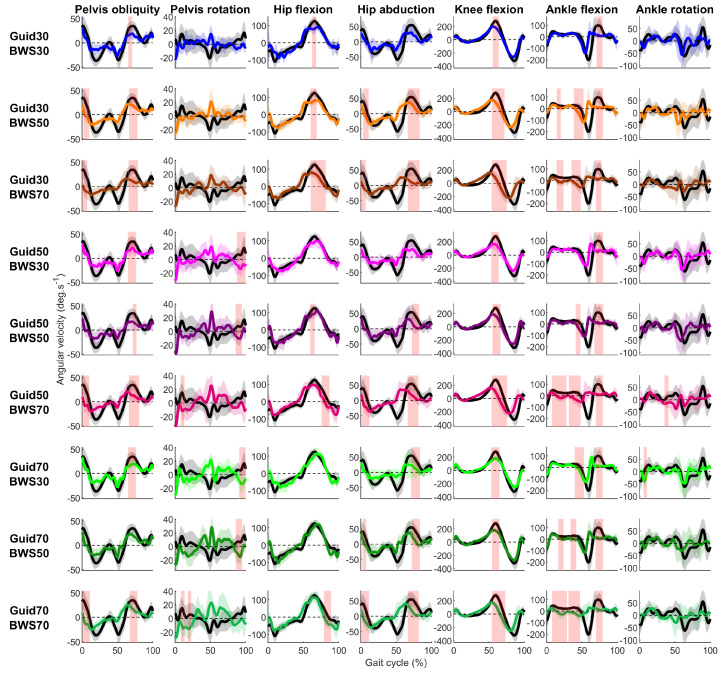
Participants’ mean (solid lines) ± standard deviation (shaded zone) joint angular velocities during the Treadmill (black) and Lokomat (colored) conditions throughout the gait cycle for each degree of freedom (column) and Lokomat setting (row). Light red shaded zones show a significant difference between the Treadmill and Lokomat conditions (FDR *p*-values are reported in Table S1).

All Lokomat settings significantly reduced participants’ stride rates compared to the Treadmill (Figure 4). In the sagittal plane, 24 of the 27 comparisons made between the Lokomat and Treadmill conditions showed significantly slower velocities for hip, knee, and ankle flexion. For the hip and knee, differences were reported around the toe-off period (Figure 5) with moderate to strong correlations (0.74 ≤ R ≤ 0.88 and 0.55 ≤ R ≤ 0.94, respectively) (Figure 3). For the ankle, differences were reported in the early swing phase for Lokomat conditions with 30% of BWS and during the stance phase for Lokomat conditions with 50% and 70% of BWS. The correlations for ankle flexion were low to moderate (0.00 ≤ R ≤ 0.42).

In the coronal and transverse planes, pelvis obliquity and hip abduction were significantly slower during the swing phase for 15 out of 18 comparisons made between the Lokomat and the Treadmill conditions (Figure 5, Table S2). Correlations were moderate to strong for pelvis obliquity (0.396 ≤ R ≤ 0.78) and low to moderate for hip abduction (0.26 ≤ R ≤ 0.58) (Figure 3). Regarding pelvic rotation, short-lasting significant differences were reported around the heel strike period between the Lokomat and the Treadmill condition for all Lokomat settings, with 50% and 70% of BWS. Correlations were low (−0.17 ≤ R ≤ 0.07).

### 3.3. Continuous Relative Phases

**Figure 6:**
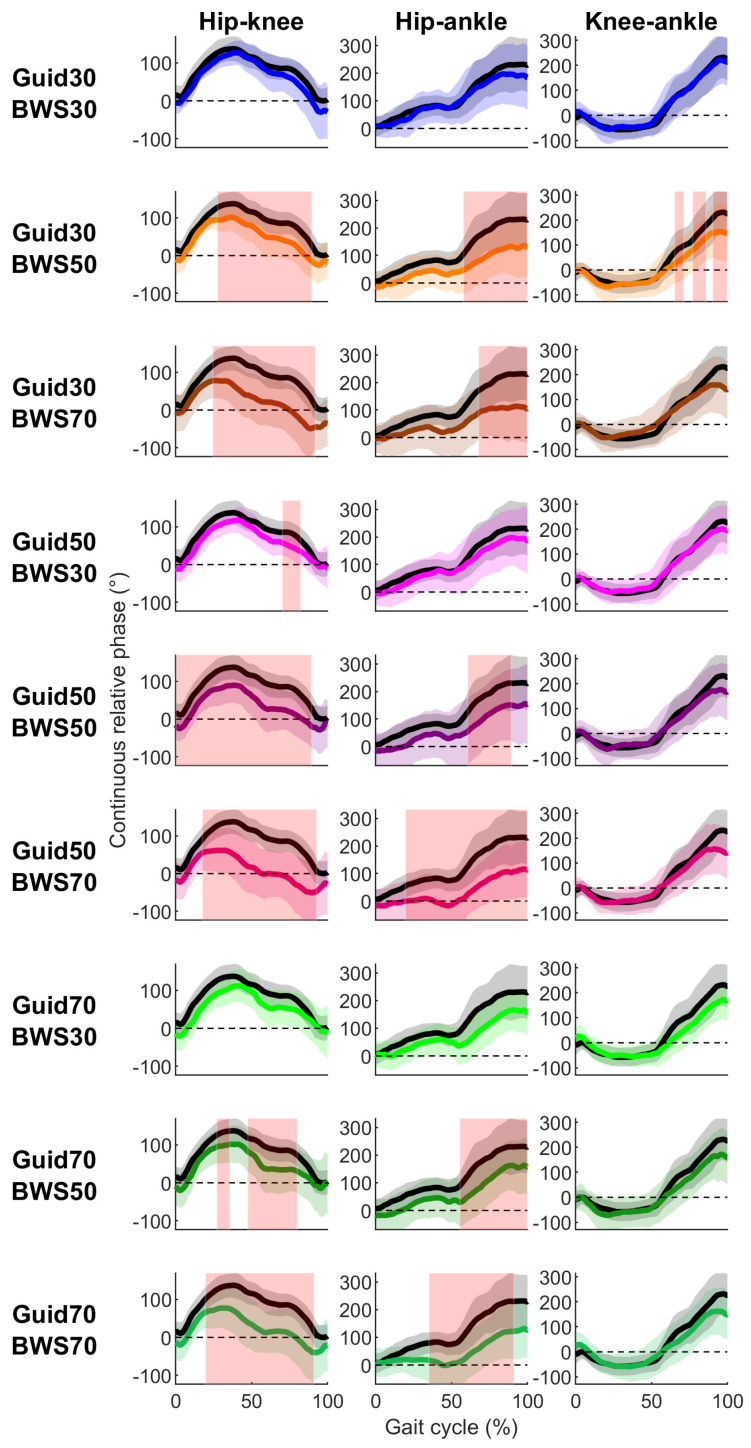
Participants’ mean (solid lines) ± standard deviation (shaded zone) CRP during the Treadmill (black) and Lokomat (colored) conditions throughout the gait cycle for each joint couple (column) and Lokomat setting (row). Light red shaded zones correspond to significant differences between the Treadmill and Lokomat conditions (FDR *p*-values are reported in Table S1).

For hip–knee and hip–ankle CRP, significant differences were reported between Lokomat and Treadmill conditions for 13 out of 18 comparisons (Figure 6, Table S3). Differences started in the early/mid-stance phase and lasted until the late swing phase (Figure 6). Moderate to strong correlations were observed between Lokomat and Treadmill conditions in terms of CRP between hip–knee (0.67 ≤ R ≤ 0.91), hip–ankle (0.66 ≤ R ≤ 0.85), and knee–ankle (0.90 ≤ R ≤ 0.95) (Figure 3).

## 4. Discussion

This study aimed to characterize the robotic-assisted Lokomat gait with different levels of BWS and Guidance in comparison to a non-assisted Treadmill gait in a healthy population. Joint angles, angular velocities, and coordination were assessed during Lokomat and Treadmill gait. Three findings warrant to be noticed. First, compared to the Treadmill, the Lokomat allowed participants to reproduce similar kinematics patterns for the hip and knee in the sagittal plane. However, walking in the Lokomat resulted in an altered pelvis rotation, reduced pelvis obliquity, and hip adduction during the stance phase compared to the Treadmill. Second, at the same walking speed, the Lokomat systematically induced a lower stride rate compared to the Treadmill. Thus, slower angular velocities were observed for the hip, knee, and ankle flexion during Lokomat conditions compared to Treadmill conditions. Third, the Lokomat modified inter-joint coordination compared to the Treadmill gait in terms of inter-joint coordination.

### 4.1. Sagittal Plane Kinematics

Hip and knee flexion showed a strong similarity between the Treadmill and the Lokomat conditions with significant differences only for the knee flexion angle during 2 of 9 Lokomat settings, namely, BWS set at 70% combined with Guidance and 30% and 50%. This result agrees with the visual inspection previously made by Hidler et al. [19] that the Lokomat, at least partially, mimics normative gait. Concerning the ankle joint, although no significant differences were found between the Lokomat and Treadmill conditions, weak correlations, especially when 70% of the body weight of the participants was supported, were reported, suggesting large inter-participant variability at this degree of freedom. This result may be explained by the ankle actuators of the Lokomat made of springs that provide less control on joint kinematics than motors actuating hips and knees. 

More unexpected results were obtained concerning angular velocity and inter-joint coordination. Indeed, although the Lokomat drives participants’ legs in the sagittal plane, hip and knee flexion/extension peak velocity around the toe-off period and hip–knee and hip–ankle coordination were modified during walking with certain combinations of settings. These results may agree with higher levels of knee flexor and extensor muscle activation during the Lokomat gait compared to the Treadmill gait [37]. Indeed, the Lokomat perturbed lower limb dynamic pattern, which is known to increase muscle activations [38]. In addition, although walking speed was constant between Lokomat and Treadmill conditions, Lokomat gait was characterized by a slower stride rate and then a lower angular velocity at the hip, knee, and ankle. This result is in line with the results of Umberger and Martin [39], which showed an increase in peak hip and knee angular velocities as the stride rate increased in healthy people walking at the same speed. Although these results indicate that the Lokomat partially replicates normative gait in the sagittal plane, improvements in hip and knee actuator controllers may be required to increase its clinical efficiency.

### 4.2. Coronal and Transverse Plane Kinematics

As expected, differences between the Lokomat and Treadmill conditions were mainly found in the coronal and transverse planes. Reduced pelvic obliquity and hip adduction, as well as an inversed pelvis rotation, were observed during the stance phase when participants walked in the Lokomat compared to the Treadmill. This result may be a direct consequence of reduced pelvis translations. Indeed, the Lokomat limits pelvis lateral displacement, which has been shown to reduce its obliquity during the stance phase [19]. Moreover, the Lokomat limits hip adduction as the cuffs set around the thigh and leg are tightened to prevent lateral movements.

Similar to the observations made for the sagittal plane, differences between the Lokomat and Treadmill gaits increased with the BWS level. This interpretation is supported by the short-lasting significant decrease in pelvis obliquity and hip adduction during experimental conditions with 30% of BWS. Although the Lokomat drives the participants’ legs only in the sagittal plane, a strong similarity was reported between the Lokomat and Treadmill gaits for pelvis obliquity and hip abduction angles, as well as pelvis obliquity angular velocity. These motions may come from the compression of the hip pads surrounding the greater trochanters, as already evidenced [2,19]. This result is observed for lesser BWS as this condition increases ground reaction forces so that larger pelvis mediolateral contact force may be applied against pads at the greater trochanters, which may increase their compression and favor pelvis obliquity.

### 4.3. Clinical Implications

Although in clinical practice the objective of the Lokomat may not be to exactly reproduce normative gait since patients may never be able to walk typically without assistance [19], some significant discrepancies between the Lokomat and the Treadmill gaits may limit the Lokomats’ clinical impact. In the sagittal plane, the hip, knee, and ankle angular velocities were slower in the Lokomat than in the Treadmill. Due to its relationship with the gait dynamics, joint angular velocities are important to be considered [40]. Joint angular velocities are, in general, reduced in patients who have spasticity due to spinal cord injury [41]. Thus, the Lokomat may not be the best strategy if the clinical objective is to increase joint excursion in spastic patients. Regarding joint coordination, the Lokomat altered hip-knee and hip-ankle coordination. However, the Lokomat accurately reproduced knee–ankle coordination. Although kinetic data on joint torque would be required to verify this interpretation, the Lokomat may adequately reproduce the plantarflexion–knee extension couple, which plays an important role in knee control during gait [42]. For example, when the plantarflexion–knee extension couple disappears, a crouch gait occurs, which is characteristic of patients with cerebral palsy [42]. Regarding the coronal plane, walking in the Lokomat reduced pelvis obliquity and hip adduction. Hip adduction plays a crucial role in controlling the mediolateral displacement of the body’s center of mass and is thus crucial for controlling dynamic balance [43,44]. The latter is often significantly impaired in people with neurologic disorders [45,46]. A combination of 30% BWS with 30% Guidance seems to be the most suitable to minimize the discrepancy between the Lokomat and Treadmill gaits in terms of hip adduction and could be relevant for patients with dynamic balance disorders.

Although there is some evidence from clinical trials that BWS and Guidance Lokomat settings are important to maximize patient benefits [47], there are currently no guidelines for best practices. This lack of guidelines and the difficulty in defining optimal settings present a major constraint in achieving the expected goals using Lokomat therapy [16,48]. Based on the present results, a combination of high levels of BWS and Guidance tended to mitigate the effect of Lokomat on lower limb kinematics. Alternatively, Lokomat conditions with 30% of BWS presented stronger similarity with Treadmill gait regardless of the Guidance level. These results are partly in agreement with previous studies showing that high levels of BWS, as well as low Guidance, result in typically low muscle activation as well as altered lower limb muscle coordination [37,49].

### 4.4. Limitations

A few limitations in this study need to be considered. Firstly, only healthy young adults were evaluated, while the Lokomat was designed for people with gait disorders. However, our results may present normative data to better characterize kinematic deviations during Lokomat gait in comparison to Treadmill gait. Secondly, the Treadmill condition was performed after the Lokomat conditions, which does not allow to exclude an after-effect of Lokomat. 

## 5. Conclusions

While the primary goal of Lokomat-based training is to enhance gait in patients with severe gait disorders, this study revealed kinematics deviations, including pelvis obliquity, rotation, and hip adduction, when comparing Lokomat to treadmill gait. These kinematics deviations were particularly pronounced under conditions with high BWS. Notably, varying levels of guidance did not have a discernible impact on gait kinematics. Our results suggest that therapists should be aware that alterations in gait patterns may occur for certain combinations of BWS and Guidance settings.

## Data Availability

Not applicable.

## Supplementary Materials

The following supporting information can be downloaded at https://www.mdpi.com/article/10.3390/s23218800/s1, Table S1: Joint angle statistical results. Intervals in the percentage of the gait cycle and in parenthesis of significant differences are shown by the statistical parametric mapping procedure between the Treadmill and each Lokomat condition. Note, n.s.: non-significant. Table S2: Angular velocity statistical results. Intervals in the percentage of the gait cycle and in parenthesis of significant differences shown by the statistical parametric mapping procedure between the Treadmill and each Lokomat condition. Note. n.s.: non-significant. Table S3: Inter-joint coordination statistical results. Intervals in the percentage of the gait cycle and in parenthesis of significant differences shown by the SPM procedure between the Treadmill and each Lokomat condition. Note. n.s.: non-significant
