# Supplementary material for: Effect of Robotic-Assisted Gait at Different Levels of Guidance and Body Weight Support on Lower Limb Joint Kinematics and Coordination
Source: Sensors (Basel). 2023 Oct 29;23(21):8800. doi: 10.3390/s23218800 (PMC10650199; doi:10.3390/s23218800)
Supplement: Supplementary file 1 [file sensors-23-08800-s001.zip › sensors-2627360-supplementary.pdf]

Supplementary materials

Table S1. Joint angle statistical results. Intervals in the percentage of the gait cycle and in parenthesis of significant differences shown by the statistical parametric mapping procedure between the Treadmill and each Lokomat condition. *Note. n.s.: non-significant.*

	Joint angles						
Conditions	Pelvis obliquity	Pelvis rotation	Hip flexion	Hip abduction	Knee flexion	Ankle flexion	Ankle rotation
Guid30 BWS30	81-89 (0.015)	13-36 (<0.001)	n.s.	41-46 (0.039)	n.s.	n.s.	n.s.
Guid30 BWS50	6-24 (<0.001)	12-47 (<0.001)	n.s.	4-53 (<0.001)	n.s.	51-56 (0.022)	n.s.
Guid30 BWS70	4-24 (0.001)	11-48 (<0.001)	n.s.	0-45 (<0.001)	72-87 (0.002)	41-57 (<0.001)	n.s.
Guid50 BWS30	4-17 (0.003)	11-46 (<0.001)	n.s.	35-52 (0.011)	n.s.	n.s.	n.s.
Guid50 BWS50	6-22 (0.004)	12-49 (<0.001)	n.s.	4-45 (<0.001)	n.s.	48-56 (0.007)	57-64 (0.02)
Guid50 BWS70	4-25 (0.005)	11-48 (<0.001)	n.s.	1-53 (<0.001)	73-88 (0.002)	39-57 (<0.001)	53-61 (0.011)
Guid70 BWS30	11-20 (0.014)	18-48 (<0.001)	n.s.	33-50 (0.004)	n.s.	n.s.	n.s.
Guid70 BWS50	10-22 (0.011)	15-49 (<0.001)	n.s.	11-53 (<0.001)	n.s.	n.s.	57-63 (0.032)
Guid70 BWS70	4-48 (<0.001)	12-49 (<0.001)	n.s.	1-52 (<0.001)	n.s.	39-57 (<0.001)	53-63 (0.006)

Table S2. Angular velocity statistical results. Intervals in the percentage of the gait cycle and in parenthesis of significant differences shown by the statistical parametric mapping procedure between the Treadmill and each Lokomat condition. *Note. n.s.: non-significant*

	Joint angular velocity						
Conditions	Pelvis obliquity	Pelvis rotation	Hip flexion	Hip abduction	Knee flexion	Ankle flexion	Ankle rotation
Guid30 BWS30	65-71 (<0.001)	n.s.	62-68 (<0.001)	n.s.	55-64 (<0.001)	70-79 (<0.001)	n.s.
Guid30 BWS50	2-10 (<0.001)<br>66-79 (<0.001)	n.s.	60-69 (<0.001)	3-11 (<0.001)<br>66-83 (<0.001)	54-72 (<0.001)	14-20 (<0.001)<br>38-52 (<0.001)<br>69-80 (<0.001)	n.s.
Guid30 BWS70	0-7 (<0.001)<br>66-79 (<0.001)	n.s.	60-82 (<0.001)	0-7 (<0.001)<br>66-83 (<0.001)	54-75 (<0.001)	14-24 (<0.001)<br>35-48 (<0.001)<br>70-78 (<0.001)	n.s.
Guid50 BWS30	65-77 (<0.001)	88-100 (<0.001)	n.s.	n.s.	53-64 (<0.001)	69-80 (<0.001)	n.s.
Guid50 BWS50	71-77 (<0.001)	86-95 (<0.001)	60-66 (<0.001)	72-82 (<0.001)	55-66 (<0.001)	41-48 (<0.001)<br>68-80 (<0.001)	n.s.
Guid50 BWS70	0-10 (<0.001)<br>66-81 (<0.001)	8-13 (<0.001)	76-87 (<0.001)	2-12 (<0.001)<br>66-81 (<0.001)	54-76 (<0.001)	7-28 (<0.001)<br>31-48 (<0.001)<br>69-80 (<0.001)	35-41 (<0.001)
Guid70 BWS30	65-76 (<0.001)	91-100 (<0.001)	n.s.	71-84 (<0.001)	53-65 (<0.001)	39-53 (<0.001)<br>70-79 (<0.001)	6-11 (<0.001)
Guid70 BWS50	n.s.	86-95 (<0.001)	n.s.	2-8 (0.001)<br>72-84 (<0.001)	54-65 (<0.001)	16-24 (<0.001)<br>34-42 (<0.001)<br>69-80 (<0.001)	n.s.
Guid70 BWS70	0-11 (<0.001)<br>67-78 (<0.001)	8-13 (<0.001)<br>18-23 (<0.001)	79-89 (<0.001)	1-12 (<0.001)<br>66-82 (<0.001)	54-73 (<0.001)	8-29 (<0.001)<br>31-47 (<0.001)<br>70-79 (<0.001)	n.s.

Table S3. Inter-joint coordination statistical results. Intervals in the percentage of the gait cycle and in parenthesis of significant differences shown by the SPM procedure between the Treadmill and each Lokomat condition. *Note. n.s.: non-significant*

	Continuous relative phase		
Conditions	Hip-Knee	Hip-Ankle	Knee-Ankle
Guid30 BWS30	n.s.	n.s.	n.s.
Guid30 BWS50	28-90 (<0.001)	58-100 (<0.001)	65-71 (0.036)<br>77-86 (0.024)<br>90-100 (0.021)
Guid30 BWS70	25-92 (<0.001)	68-100 (<0.001)	n.s.
Guid50 BWS30	70-82 (0.006)	n.s.	n.s.
Guid50 BWS50	0-89 (<0.001)	61-89 (0.001)	n.s.
Guid50 BWS70	18-93 (<0.001)	20-100 (<0.001)	n.s.
Guid70 BWS30	n.s.	n.s.	n.s.
Guid70 BWS50	27-35 (0.027)<br>47-80 (<0.001)	55-100 (<0.001)	n.s.
Guid70 BWS70	20-91 (<0.001)	35-91 (<0.001)	n.s.
